# Menstrual blood-derived mesenchymal stromal cell secretome modulates macrophage polarization in a preconditioning-dependent manner

**DOI:** 10.3389/fcell.2025.1691010

**Published:** 2026-01-22

**Authors:** María Ángeles de Pedro, María Pulido, Ana María Marchena, Verónica Álvarez, Francisco Manuel González-Nuño, Witold Szymański, Johanna Pörschke, Silke Reinartz, Johannes Graumann, Elke Pogge von Strandmann, Francisco Miguel Sánchez-Margallo, María Gómez-Serrano, Esther López

**Affiliations:** 1 Stem Cell Therapy Unit, Jesús Usón Minimally Invasive Surgery Centre, Cáceres, Spain; 2 RICORS-TERAV Network, ISCIII, Madrid, Spain; 3 Institute of Translational Proteomics, Biochemical/Pharmacological Center, Philipps-Universität, Marburg, Germany; 4 Core Facility Translational Proteomics, Philipps-Universität, Marburg, Germany; 5 Institute for Tumor Immunology, Center for Tumor Biology and Immunology (ZTI), Philipps-Universität, Marburg, Germany; 6 Translational Oncology Group, Center for Tumor Biology and Immunology (ZTI), Philipps-Universität, Marburg, Germany; 7 EV-iTEC Core Facility, Philipps-Universität, Marburg, Germany

**Keywords:** macrophage polarization, menstrual blood-derived mesenchymal stromal cells, mesenchymal stromal cells, secretome, stem cell therapy

## Abstract

**Background:**

The effects of menstrual blood-derived mesenchymal stromal cell secretome (S-MenSC) on macrophage polarization remain unclear. This study studied the impact of secretomes from basal MenSCs (S-bMenSCs) and those preconditioned with IFNγ and TNFα (S-pMenSCs) on human monocytes and macrophages *in vitro*.

**Methods:**

S-MenSCs were used to assess their effects on three stages of monocyte-derived cell maturation: i. monocyte differentiation; ii. polarization of monocyte-derived macrophages (MDMs) toward M1-like or M2-like phenotypes; and iii. reprogramming of pre-polarized M1 or M2 macrophages. Surface markers were analyzed by flow cytometry and cytokine gene expression by RT-qPCR. In addition, a proteomic profiling was performed to identify proteins involved in the observed effects.

**Results:**

Our results confirmed the capacity of S-MenSCs of modulating innate immune response and in particular macrophage polarization. More concretely, the *in vitro* experiments showed that: i. both secretomes partly promoted monocyte differentiation into an M1-like phenotype; ii. during macrophage polarization, S-bMenSCs partially limited the shift to an M1 phenotype, whereas treatment with S-pMenSCs boosted it; and, iii. in the pre-polarized macrophages, S-bMenSCs reinforced M1 traits, whereas S-pMenSCs promote partial phenotype switching. Finally, proteomic analysis revealed significant differences in the composition of both secretomes, comprising key proteins associated with macrophage polarization.

**Conclusion:**

These findings extend the knowledge on the immunomodulatory capacity of the S-MenSC, supporting that MenSCs, particularly when preconditioned, may play a significant role in regulating macrophage polarization, and, thus, modulating the inflammatory response.

## 1 Introduction

In recent years, alternative sources of mesenchymal stromal cells (MSCs) have become a major focus in regenerative medicine and immunotherapy. Among other MSC sources (e.g., bone-marrow, umbilical-cord), menstrual blood-derived MSCs (MenSCs) have emerged as a promising candidate for clinical applications (Galea et al., 2022). MenSCs not only share characteristics with traditional MSCs, but also exhibit unique advantages, including extremely easy and non-invasive isolation, free of ethical concerns, as well as an enhanced proliferative capacity (Lv et al., 2018; Bozorgmehr et al., 2020). While immunomodulatory and regenerative properties are shared by many MSC types, MenSCs combine these capacities with distinctive features that may further support their therapeutic potential (Chen et al., 2019a; Chen et al., 2019b). Previous research has demonstrated that MenSCs may regulate the immune response by interacting with various immune cells, including macrophages (Bozorgmehr et al., 2020; Chen et al., 2019b), through the secretion of regulatory molecules and cytokines (Lv et al., 2018). Additionally, MenSCs have shown strong regenerative potential, promoting tissue repair in a range of experimental models (Galea et al., 2022; Bozorgmehr et al., 2020). Therapeutic applicability has been demonstrated in immune- and inflammation-based diseases, including macrophage-mediated disorders like thioglycollate-induced peritonitis or monobacterial sepsis (Martinez-Aguilar et al., 2020), as well as in clinical trials for type I diabetes (NCT01496339) or COVID-19 (NCT05019287). Cell therapy based on MSCs, however, remains controversial (Amadeo et al., 2021). For instance, MSCs have demonstrated relatively low survival rates after administration, and their paracrine activity is unpredictable due to strong influences of the receiving microenvironment (Peshkova et al., 2023). To address these challenges, several strategies are being explored, including the encapsulation of MSCs in hydrogels or scaffolds (Raghav et al., 2022) as well as the use of their secretome or extracellular vesicles (EVs), among others. EVs, in particular, offer multiple advantages, including enhanced stability, consistent biological activity, and the potential for freeze-drying (Raghav and Mann, 2024). However, it has been shown that their therapeutic efficacy is amplified by synergistic interactions with proteins and other components present in the complete secretome (Mitchell et al., 2019; Wong et al., 2025). Within this context, the secretome of MSCs, rather than the cells themselves, emerges as a possibly more reliable and standardizable tool, as the therapeutic potential of MSCs is largely attributed to their secretome (Chen et al., 2021). Exploring this strategy for the development of effective cell-free therapy, this work explores the specific components involved and the potential mechanisms of action of the MenSC-derived secretome.

Recent studies have demonstrated that the cross-talk between MSCs and macrophages to be essential for the immunomodulatory and regenerative therapy mediated by MSCs (Peshkova et al., 2023; Ti et al., 2024). Macrophages, as part of the first line of defense in the immune system, are actively involved throughout inflammatory processes. They are known to polarize into distinct functional phenotypes, traditionally classified as M1 (pro-inflammatory) and M2 (anti-inflammatory), and this polarization capacity is crucial for regulating the immune response, as well as for the maintenance of the balance between inflammation and tissue repair (Ti et al., 2024; Lu et al., 2021). It is further accepted that exposure to pro-inflammatory stimuli such as interferon gamma (IFNγ), lipopolysaccharide (LPS), or tumor necrosis factor alpha (TNFα) favors the shift toward an M1-like classically activated phenotype (Kerneur et al., 2022). M1 macrophages exhibit increased ability for antigen presentation, phagocytic activity, and the production of chemokines. By releasing inflammatory mediators, M1 macrophages promote the elimination of intracellular pathogens and play a crucial role in tumor prevention (Lu et al., 2021). In contrast, M2 macrophages are associated with anti-inflammatory contexts and tissue repair. These cells are further subclassified into M2a, M2b, and M2c subtypes, each induced by different signals and performing distinct functions (Lu et al., 2021). In this study, we specifically focused on M2c macrophages, which are induced by glucocorticoids and IL10 (Holthaus et al., 2022). This macrophage subtype has been implicated in suppressing exacerbated immune responses and in the resolution of inflammation, playing an important role in maintaining homeostasis in chronic inflammatory contexts as well as in tissue remodeling after injury (Holthaus et al., 2022). It is important to note that the classification of macrophages into M1 and M2 represents a conceptual model that helps to study the different functional states of these cells under experimental *in vitro* conditions. This definition is, however, oversimplified, as in a physiological setting, macrophages do not exhibit such clearly defined polarized states (Strizova et al., 2023).

MSCs from various sources have been shown to modulate macrophage polarization, often promoting a shift toward an M2-like phenotype (Peshkova et al., 2023; Luz-Crawford et al., 2017). MSCs suppress both the transition of human macrophages from the unpolarized state (*i.e.*, M0) to the M1-like state, as well as the pro-inflammatory activity of macrophages already polarized to M1-like state (Peshkova et al., 2023). However, the effect of the MenSC-derived secretome, the promising new source of MSCs, on macrophage polarization remains largely unexplored. Gaining a deeper understanding of this interaction may result in the development of novel therapeutic strategies targeting immunological or inflammation-based diseases. We previously demonstrated that the particular combination of IFN-γ and TNF-α synergistically enhances the release of IDO1 (De Pedro et al., 2021), also influencing the extracellular vesicle (EV) component of the secretome (de Pedro et al., 2023). Given these insights, the objective of this study was to assess the immunomodulatory capacity of the secretome of MenSCs in terms of macrophage differentiation, polarization and repolarization toward a pro- or anti-inflammatory phenotype, both in the presence and absence of pro-inflammatory pre-stimuli. In this work, we focused on the whole protein compartment of the secretome through mass-spectrometry analyses in order to identify the potential immunomodulatory molecules released by MenSCs, extending our previous characterization at the miRNA (De Pedro et al., 2021) and EV level (De Pedro et al., 2023). To our knowledge, this work represents the first attempt to characterize and dissect the impact of the MenSC-derived secretome under basal and after preconditioning with IFNγ and TNFα on the polarization of monocytes and macrophages. Our findings revealed that preconditioning strategies effectively modulate the proteome composition of the MenSC-derived secretome, and in contrast to other MSC sources, promote a shift toward a pro-inflammatory macrophage phenotype.

## 2 Methods

### 2.1 MenSCs culture and secretome collection

MenSCs were obtained from the cell bank of the Stem Cell Therapy Unit at the Jesús Usón Center for Minimally Invasive Surgery. These cells were isolated as previously described (Alvarez et al., 2018), including five healthy female donors with a mean age of 30.2 ± 3.1 years. All donors had regular menstrual cycles, were not using hormonal contraceptives, and had no known gynecological or systemic diseases. Briefly, MenSCs were cultured in Dulbecco’s Modified Eagle Medium - High Glucose (DMEM) with 10% fetal bovine serum (FBS, GibcoTM, Thermo Fisher Scientific, Bremen, Germany), 1% penicillin/streptomycin, and 1% glutamine (Thermo Fisher Scientific, MA, United States) at 37 °C and 5% CO2. Experiments were conducted between passages P4-P8 and at 80% confluence. Before secretome collection, MenSCs were pretreated with a pro-inflammatory stimulus of IFNγ and TNFα (both at 100 ng/mL, Miltenyi Biotec, CA, United States) for 72 h, which has been previously demonstrated to enhance the immunomodulatory capacity of MenSCs (de Pedro et al., 2021). After pretreatment of MenSCs, cells (basal and primed) were washed with PBS 1x and cultured in FBS-free DMEM High Glucose supplemented with 1% insulin-transferrin-selenium (ITS) (Cat. No. 41400045, Thermo Fisher Scientific) for 48 h. Of note, ITS is an essential factor for MenSC survival under serum-free conditions (Liu et al., 2019). Vitality, metabolic activity and apoptosis of MenSCs during the course of secretome collection was assessed (Supplementary Figure S1). The conditioned media (14 mL per 175 cm^2^ flask) were collected and first centrifuged at 1000 g 10 min at 4 °C, followed by a second centrifugation at 5000 g 20 min at 4 °C. The supernatants were then filtered through 0.45- and 0.22-µm filters to remove cellular debris, thereby obtaining the corresponding secretomes. Secretome solutions were concentrated up to a final volume of 400 µL (35 times v/v) using a 3 kDa MWCO Amicon® Ultra device (Cat No. UFC900324, Merck-Millipore, MA, United States) by centrifugation at 4000 g for 40 min at 4 °C. Following, the secretome protein concentration was quantified using a Bradford assay (BioRad, CA, United States), according to the manufacturer’s instructions. Considering the cultivation surface area, the estimated average protein yield of the MenSC secretome after concentration was approximately 5 μg/cm^2^ for basal secretomes and 4 μg/cm^2^ after priming. Finally, the secretomes from basal, non-treated MenSCs (S-bMenSCs) and from MenSCs primed with IFNγ and TNFα (S-pMenSCs) were subsequently aliquoted and stored at −80 °C until further use.

### 2.2 Human monocyte isolation, differentiation and monocyte-derived macrophage (MDM) polarization

Leukoreduction System (LRS) chambers with 10 mL thrombocyte and leucocyte enriched blood from healthy adult volunteers were provided by the Center for Transfusion Medicine and Hemotherapy at the University Hospital Gießen and Marburg (UKGM). Materials were used with the informed consent of the donors and approved by the local ethics committee (No. 05/00). Mononuclear cells were isolated by Ficoll density gradient centrifugation. Subsequently, monocytes were purified by adherence selection and used for differentiation in RPMI 1640 (Gibco™) supplemented with 5% human AB serum (Cat. MFCD00165829, Sigma-Aldrich, Taufkirchen, Germany) and 1 mM sodium pyruvate (Sigma-Aldrich) at approximately 2.5 × 10^6^ cells per well in 6-well plates. To minimize the potential impact of heterogeneity among MenSC donors, a pooled sample comprising equal contributions from five donors was created to evaluate the overall effects of the MenSC-derived secretome rather than those of individual donors. These pooled samples were used to assess the effects of S-MenSCs in three distinct experimental settings. First, to address the effect of S-MenSC on monocyte differentiation (n = 3 donors), after adherence, the cells were allowed to differentiate for 7 days in the presence of S-MenSCs (final concentration of 100 μg/mL). No media renewal was performed over the course of the experiment, and monocytes cultured in medium were used as a reference control. Second, for M1-like or M2-like polarization assays (n = 6 donors), 100 ng/ml GM-CSF (Cat. 300-03, PeproTech®, Thermo Fisher Scientific) or 20 ng/ml M-CSF (Cat. 574804, BioLegend, CA, United States) were added to adherent monocytes to differentiate them into M1-like or M2-like monocyte-derived macrophages (MDMs), respectively. Pre-commitment of MDMs (n = 3 donors) was achieved for 5 days with GM-CSF (100 ng/mL), IFNγ (20 ng/mL, Cat. 51564.100, Biomol, Hamburg Germany), and LPS (100 ng/mL, Cat. L439, Sigma-Aldrich) for M1-like and M-CSF (20 ng/mL) and IL10 (20 ng/mL, Cat. 1145, Proteintech HZ, Manchester, United Kingdom) for M2-like, respectively. After 5 days, half of the medium was renewed and S-MenSC treatments were applied (final concentration 100 μg/mL) simultaneously with the corresponding cytokines. MDMs were detached for flow cytometry (FC) and RNA collection on day 7. Lastly, for post-polarization experiments, MDMs were differentiated and polarized for 7 days as described (without the input of secretome). After polarization, MDMs were thoroughly washed with PBS to remove residual stimuli and then treated exclusively with S-MenSCs (final concentration 100 μg/mL) for 48 h, followed by analysis of polarization markers. In all cases, untreated MDMs were used as the reference group. Secretome pools were adjusted to a final concentration of 100 μg/mL total protein and then used at this concentration for all treatments. Potential effects of secretome collection medium on monocytes and MDMs were additionally evaluated in a volume-based manner, meaning that ITS was applied at the same volume as the other two secretome treatments (Supplementary Figure S2).

### 2.3 Analysis of surface markers by flow cytometry

To analyze the phenotype of monocytes or MDMs, FC analyses were performed using specific surface antibodies against the typical M1-like (CD80, CD86) and M2-like (CD163, CD206) markers. MDMs were detached using TrypLE (Gibco^TM^), blocked for 1 h at 4 °C to prevent non-specific binding (1:100 Mouse-Gamma-Globulin, Jackson Research, GA, United States), and stained with fluorochrome-conjugated monoclonal antibodies for 30 min at 4 °C in the dark. Primary antibodies used were CD80-PE (Cat. 12-0809-42, Clone 2D10.4, eBioscience, Thermo Fisher Scientific), and CD86-FITC (Cat. 130-116-159, Clone REA968, Miltenyi Biotec, North Rhine-Westphalia, Germany), CD206-APC (Cat. 321110, Clone 15-2, Biolegend), CD163-PE-Cy7 (Cat. 12-1639-42, Clone GHI/6, eBioscience), in addition to their corresponding isotype controls: mouse IgG2α-FITC (Cat. 130-113-271, Clone S43.10, Miltenyi Biotec), mouse IgG1κ-PE (Cat. 12-4714-42, Clone P3.6.2.8.1, eBioscience), mouse IgG1κ-PE-Cy7 (Cat. 15-4714-81, Clone P3.6.2.8.1, eBioscience), and mouse IgG1-APC (Cat. 400120, Clone MOPC-21, Biolegend). FC analyses were conducted using a BD FACSCanto^TM^ II (BD Biosciences, San Jose, CA). Data acquisition and analysis were performed using FlowJo software V10.8.1. The full gating strategy is provided in the Supplementary Material (Supplementary Figure S3). In summary, debris and aggregates were excluded by conservative FSC-A vs. SSC-A gating, followed by quantitative filtering to remove events with FSC-A > 99th percentile and SSC-A < 1st percentile, generating the filtered cell population. The total population of macrophages was analyzed directly within the filtered cell population. Marker positivity was defined using corresponding fluorescence minus one (FMO) controls, setting the threshold at the 98–99th percentile of the FMO distribution. Both frequency (percentage of positive cells) and geometric mean fluorescence intensity (GeoMFI) were calculated for each marker. Moreover, all raw FC data have been deposited in Zenodo under the following DOIs: 10.5281/zenodo.17522320, 10.5281/zenodo.17540731, 10.5281/zenodo.17541002, 10.5281/zenodo.17541055, and 10.5281/zenodo.1754155, corresponding to the experimental datasets for monocytes, MDM-M1-like, MDM-M2-like, post-polarized M1 macrophages, and post-polarized M2 macrophages, respectively.

### 2.4 Cytokine expression analyses

Quantitative real-time PCR (RT-qPCR) was used to evaluate the mRNA expression levels of several cytokines in monocytes or MDMs. Previously, total RNA was extracted from the cells using the NucleoSpin^R^ RNA II isolation kit (Macherey-Nagel, Nordrhein-Westfalen, Germany) according to the manufacturer’s instructions. RNA concentration and purity were measured using a Nanophotometer NP80 (Implen, München, Germany). Complementary DNA (cDNA) was synthesized from 100 ng of total RNA using iScript™ Reverse Transciption Supermix (BioRad, CA, United States). Lastly, RT-qPCR was performed using TaqMan® Fast Advance Master Mix and TaqMan gene expression assays (Applied Biosystems) on a QuantStudio™ 3 Real-Time PCR System (Applied Biosystems, Thermo Fisher Scientific, MA, United States) and analyzed with Thermo Fisher Cloud software using the 2^−ΔΔCT^ method. The following TaqMan probes were utilized for gene expression analysis: *TNF* (hs00174128_m1), *IL6* (hs00174134_m1), *IL1B* (hs01555410_m1), *TGFB* (hs00998133_m1), *IL10* (hs00961622_m1), and *TBP* (hs00427620_m1), the latter as reference gene. All assays were performed in technical duplicates.

### 2.5 Sample preparation and digestion for proteomic analyses

100 μL of secretome was obtained from five individual samples under basal (S-bMenSC) and preconditioned conditions (S-pMenSC) (n = 5 per group). To each volume of conditioned media, 2% deoxycholic acid sodium salt (DOC) solution was added to a final concentration of 0.02% and incubated at room temperature for 15 min. Then, a 100% trichloroacetic acid (TCA) solution was added to a final concentration of 10%. The solution was mixed and incubated at room temperature for 1 h. Subsequently, samples were centrifuged for 10 min at maximum speed, the supernatant was removed, and the pellet retained. 200 μL of ice-cold acetone was added to the protein pellet, mixed, and incubated on ice for 15 min, followed by centrifugation for 10 min at maximum speed at 4 °C and discarding of the supernatant. Pellets were resuspended in 50 μL of a 4% sodium lauroyl sarcosinate (SLS) solution and incubated at 95 °C for 10 min. Peptide concentration was estimated via BCA Protein Assay, and the volume corresponding to 50 μg of protein used for further processing. Reduction and alkylation were performed by adding dithiothreitol (DTT) to a final concentration of 10 mM with incubation at 95 °C for 10 min, followed by iodoacetamide (IAA) at a final concentration of 13 mM with incubation at 25 °C for 30 min. A modified SP3 protocol (Hughes et al., 2019) was used for sample preparation on a custom magnetic rack. Proteins were bound in a solution containing 70% anhydrous acetonitrile (ACN) at neutral pH, followed by washing with 70% ethanol and 100% ACN. After removing ACN, beads were resuspended in 50 μL of 50 mM TCA buffer, and 1 μg of trypsin (Promega, Madison, Wisconsin, United States) was added. Proteins were digested overnight at 37 °C. Post-digestion, sample volumes were reduced to approximately 5 μL using a SpeedVac concentrator (Thermo Fisher Scientific). Peptides were rebound to beads by adding 100% ACN to a final concentration exceeding 98%. Beads were washed twice with ACN, and peptides were eluted using 40 μL of 0.1% formic acid (FA) before being transferred to sample vials. Peptide concentration was quantified using the fluorometric Pierce Quantitative Peptide Assay, and sample volumes were adjusted to ensure equal concentration across all samples.

### 2.6 Mass spectrometry acquisition and spectra identification

Purified peptides were analyzed by liquid chromatography–tandem mass spectrometry (MS) carried out on a Bruker Daltonics timsTOF Pro instrument connected to a Bruker Daltonics nanoElute instrument. Approximately 300 ng of peptides were loaded onto a C18 precolumn (Thermo Trap Cartridge 5 mm, μ-Precolumn™ Cartridge/PepMap™ C18, Thermo Scientific) and eluted in backflush mode with a gradient from 98% solvent A (0.15% FA) and 2% solvent B (99.85% ACN and 0.15% FA) to 17% solvent B over 36 min, continued from 17% to 25% solvent B over 18 min, then from 25% to 35% solvent B over another 6 min on a reverse-phase high-performance liquid chromatography (HPLC) separation column (Aurora Series Emitter Column with CSI fitting, C18, 1.6 μm, 75 μm × 25 cm, Ion Optics) with a flow rate of 400 nL/min. The outlet of the analytical column with a captive spray fitting was directly coupled to the MS instrument. Data were acquired using a data-independent acquisition (DIA) paradigm using a default method provided by Bruker. In short, spectra were acquired with a fixed resolution of 45,000 and a mass range from 100 to 1700 m/z for the precursor ion spectra and an a 1/k0 range from 0.6 to 1.6 V s/cm^2^ with a 100 ms ramp time for ion mobility, followed by DIA scans with 21 fixed DIA windows of 25 m/z width, ranging from 487.5 to 1012.5 m/z.

Peptide spectrum matching and label-free quantitation were subsequently performed using DIA-NN 1.8.1 (Demichev et al., 2020) in a library-free search against the Human Uniprot.org database (20,429 reviewed Swiss-Prot entries; January 2024). In brief, output was filtered to a 1% false discovery rate (FDR) at the precursor level. Deep learning was used to generate an *in silico* spectral library for the library-free search. Fragment m/z was set to a minimum of 200 and a maximum of 1,800. In silico peptide generation allowed for N-terminal methionine excision, tryptic cleavage following K*, R*, a maximum of one missed cleavage, as well as a peptide length requirement of seven amino acids minimum and a maximum of 30. Cysteine carbamidomethylation was included as a fixed modification and methionine oxidation (maximum of two) as a variable modification. Precursor masses from 300 to 1800 m/z and charge states up to four were considered. DIA-NN was instructed to optimize mass accuracy separately for each acquisition analyzed, and protein sample matrices were filtered using a run-specific protein q-value (“--matrix-spec-q” option).

### 2.7 Bioinformatic data processing and statistical analysis

Downstream data processing and statistical analysis were carried out using the *Autonomics* package developed in-house (Version 1.11.33) (Bhagwat and Graumann, 2025). Briefly, peptide/spectrum-matching using DIA-NN was filtered to a 1% FDR at the precursor level and only proteins groups with a *q* value <0.01 were retained for further analysis. Concretely in our study, we initially identified 3698 protein groups. MaxLFQ (Cox et al., 2014) values were used for quantitation, and missing values were imputed. All intensities and MaxLFQ values that contain only one precursor per sample were replaced by NA for that particular sample. After dropping 13 without replication (within the subgroup) and filtering out 686 proteins with fewer than 2 peptides identified, 2,999 protein groups were retained for further analysis. Differential abundance of protein groups between primed (S-pMenSC) and basal (S-bMenSC) secretomes was evaluated by *Autonomics* employing a Bayesian moderated t-test as implemented by limma (Ritchie et al., 2015). The full list of DIA-NN settings can be found in “report.log.txt”; code for data processing and statistical analysis can be found in “ag_pogge-serrano-mdp-tams-001.qmd”, uploaded, along with the mass spectrometric raw data and UniProt database, to the ProteomeXchange Consortium with dataset identifier: PXD060067, via the MassIVE partner repository (https://massive.ucsd.edu/, MassIVE-ID: MSV000096909; doi:10.25345/C5NZ81269).

Functional and pathway enrichment analysis were performed on the top 20% most abundant proteins identified (based on normalized MaxLFQ values) using Enrichr (https://maayanlab.cloud/Enrichr/). The 10 most significantly enriched terms were visualized using bar plots created with the ggplot2 package in RStudio (version 4.3.2). Principal component analysis (PCA) was conducted to examine variability between basal (S-bMenSCs) and primed (S-pMenSCs) secretomes. Differentially abundant proteins (DAPs) were identified using volcano plots, applying a threshold of log2FC = 1 and an adjusted p < 0.05, subsequent to FDR correction for multiple hypothesis testing. Additionally, the presence and abundance of proteins associated with macrophage polarization were evaluated in the secretomes.

Statistical analyses were performed using GraphPad Prism (version 8.0). For each marker, data consisted of paired measurements obtained from the same donors under three experimental conditions (control, basal, and primed). For experiments with n = 6 donors, normality of residuals was assessed using the Shapiro–Wilk test. Markers meeting normality assumptions were analyzed with repeated-measures one-way ANOVA followed by Tukey’s *post hoc* test, whereas non-normal data were analyzed using the non-parametric Friedman test with Dunn’s multiple comparisons. For experiments with n = 3 donors, normality could not be reliably assessed due to the small sample size; therefore, all analyses were performed using the non-parametric Friedman test with Dunn’s *post hoc* correction. Data are presented as mean ± SEM, and individual donor values are indicated in all graphs with different symbols. Statistical significance was defined as p-value ≤0.05.

## 3 Results

### 3.1 Functional insight from the proteome of the MenSC secretome

**FIGURE 1:**
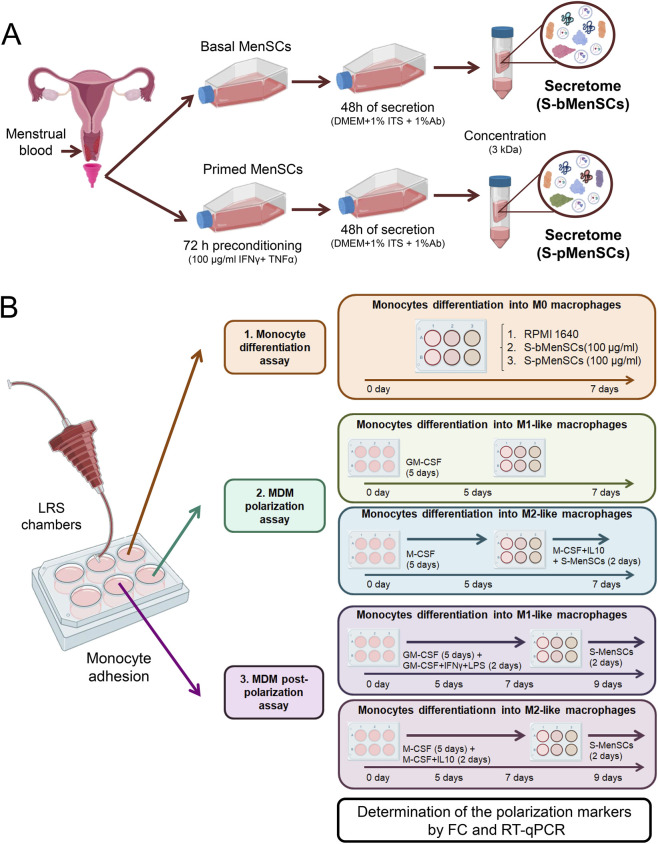
Study design. **(A)** Diagram of secretome collection. **(B)** Monocyte-derived macrophage (MDM) assays: monocyte differentiation assay (1), MDM polarization assay (2), and MDM post-polarization assay (3). Both secretome treatments (basal, S-bMenSCs; and primed, S-pMenSCs) were applied at different time-points at a final concentration of 100 µg/ml. Untreated cells were used as a negative control. Polarization markers were assessed by flow cytometry (FC) and RT-qPCR at the end of each assay. Ab, antibiotics (penicillin/streptavidin); ITS, Insulin-Transferrin-Selenium; MenSC, menstrual blood-derived stromal cells; S-MenSCs, secretome released by menstrual blood-derived stromal cells. This figure was partially created with BioRender, https://app.biorender.com/.

**FIGURE 2:**
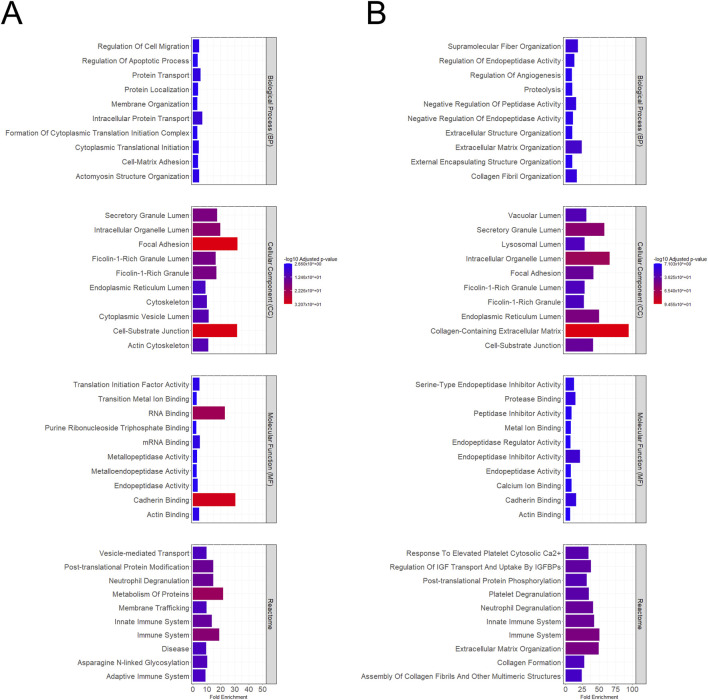
Functional insights of the S-MenSC proteome. The top 20% most abundant proteins identified in the **(A)** S-bMenSC (n = 567 protein groups) and **(B)** S-pMenSC (n = 560 protein groups) were categorized according to the *Gene Ontology* (GO) terms *Biological Processes* (BP), *Cellular Components* (CC), and *Molecular Functions* (MF), as well as *Reactome pathway*s, and presented as bar graphs (fold enrichment). Color intensity indicates and scaled -log10 (enrichment *p value*). Protein lists are depicted in Supplementary Tables S1–S4. S-bMenSCs, secretome released by basal menstrual blood-derived stromal cells; S-pMenSCs, secretome released by menstrual blood-derived stromal cells pretreated with IFNγ and TNFα.

This work aimed to evaluate the functional effects of the MenSC-derived secretome (Figure 1A) in a clinically relevant *in vitro* model, namely human monocyte-derived macrophages (MDMs) (Figure 1B). We first examined the protein content of the secretome in terms of their overall abundance and potential functional implications. The secretory proteome profile of basal MenSCs (S-bMenSCs), revealed that the top 20% of the most abundant proteins (Supplementary Table S1) were significantly involved in *Biological Processes* (BP) associated with molecular transport and structural organization according to *Gene Ontology* (GO) enrichment analyses (Figure 2A; Supplementary Table S2). In addition, processes linked to cell migration and adhesion were prominent. In terms of *Cellular Components* (CC), the proteins were predominantly linked to intracellular trafficking (e.g., *Cytoskeleton*) and cellular communication networks (e.g., *Focal Adhesion* and *Cell-Substrate Junction*). The analysis of the *Molecular Function* (MF) further indicated involvement of the S-MenSC in cellular maintenance, regulation, and response processes (e.g., *cadherin* and *actin binding*, *RNA* and *mRNA binding*, and *Metallopeptidase Activity*, respectively). Finally, *Reactome pathway* analyses of the S-bMenSC proteome highlighted their engagement in immune-related processes, particularly pathways associated with innate immune system activation and cell signaling events (e.g., *Vesicle-mediated transport and membrane trafficking*) (Figure 2A). In the case of the pre-conditioned MenSCs, the proteome profile of their derived secretome (S-pMenSCs) (Supplementary Table S3), presented a significant enrichment in BP related to extracellular matrix (ECM) organization and regulation of proteolytic activity, reflecting a potential role in tissue structure maintenance and remodeling. In line with these observations, proteins related to the regulation of angiogenesis were also significantly enriched (Figure 2B; Supplementary Table S4). Enrichment analyses for CC included *collagen-containing ECM*, *focal adhesions*, and various intracellular organelle lumen proteins, emphasizing the hypothesized role in intercellular communication and maintenance of structural integrity. In terms of MF, key activities included enzyme inhibition and structural protein binding (e.g., *Peptidase Inhibitor Activity* and *Actin Binding*), crucial for regulating protease activity and supporting cell adhesion. *Reactome* analysis of the S-pMenSCs further underscored pathways involved in immune system regulation, including those related to innate immunity, neutrophil degranulation, and platelet degranulation (Figure 2B; Supplementary Table S4). These findings suggested that S-pMenSCs may play a substantial role in modulating immune responses, as for S-bMenSCs, while also contributing to ECM formation and collagen assembly, which are essential processes for tissue repair and remodeling.

### 3.2 Effect of the S-MenSC secretomes on monocyte differentiation

**FIGURE 3:**
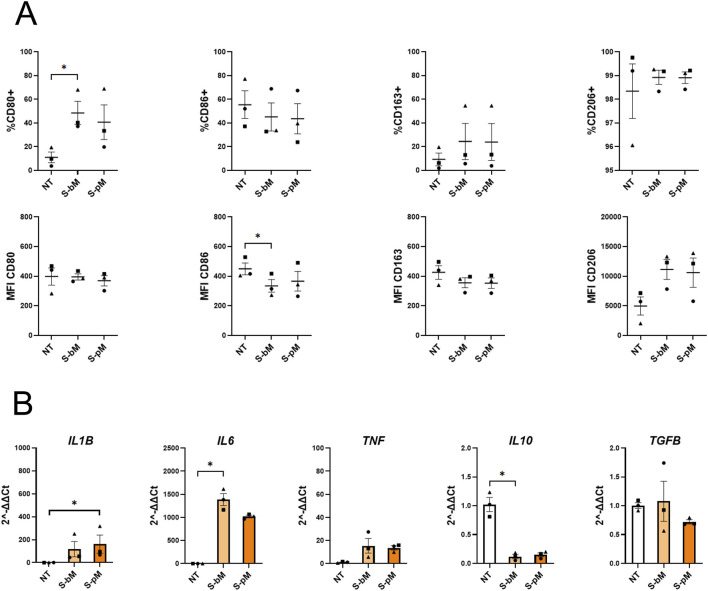
Effect of the S-MenSC secretomes on monocyte differentiation. Monocytes were differentiated into MDMs for 7 days in the presence of basal (S-bMenSC) or IFNγ/TNFα-primed (S-pMenSC) secretomes. **(A)** Flow cytometry analysis of M1-associated markers (CD80, CD86) and M2-associated markers (CD163, CD206), represented as both the frequency of positive cells (% of cells) and the geometric mean fluorescence intensity (GeoMean). **(B)** RT-qPCR analysis of pro-inflammatory cytokines (*IL1B, IL6, TNF*) and anti-inflammatory cytokines (*IL10, TGFB*). Data are presented as mean ± SEM. Statistical significance was determined using the Friedman test with Dunn’s multiple comparisons (n = 3): *, p < 0.05; **, p < 0.005. NT, non-treated monocytes; S-bM, monocytes treated with basal MenSC secretome; S-pM, monocytes treated with primed MenSC secretome.

To investigate whether S-MenSCs were able to alter monocyte differentiation into MDMs, isolated monocytes were treated exclusively with S-bMenSCs or S-pMenSCs (no additional cytokines) and analyzed by FC and RT-qPCR for markers associated with M1/M2 phenotypes after 7 days (Figure 3). Overall, our results indicated a shift towards a more M1-like phenotype upon S-MenSC stimuli, regardless of the secretome type. More specifically, CD80 expression increased under both secretome treatments, reaching statistical significance with S-bMenSCs and showing a similar upward trend with S-pMenSCs, although not significant (Figure 3A). This was accompanied by elevated levels of *IL1B*, *IL6*, and *TNF*, with *IL6* being significant under S-bMenSC treatment and *IL1B* significant under S-pMenSC treatment (Figure 3B), indicating a clear promotion of an M1-like pro-inflammatory phenotype. CD86 expression was reduced under both treatments, achieving significance with S-bMenSCs (Figure 3A), further supporting an attenuated anti-inflammatory or M2-like state. Regarding classical M2-associated markers, *IL10* expression decreased under both treatments, reaching statistical significance with S-bMenSCs (Figure 3B), while CD163 and CD206 showed non-significant upward trends. Together, these data suggest that both treatments may promote a regulated inflammatory response characterized primarily by monocyte differentiation towards an M1-like pro-inflammatory phenotype (*i.e.,* high IL1B or *IL6*, low *IL10* expression), with a modest and non-significant induction of certain M2 markers, possibly reflecting a controlled or balanced inflammatory activation.

### 3.3 Effect of MenSC-derived secretomes on macrophage polarization

**FIGURE 4:**
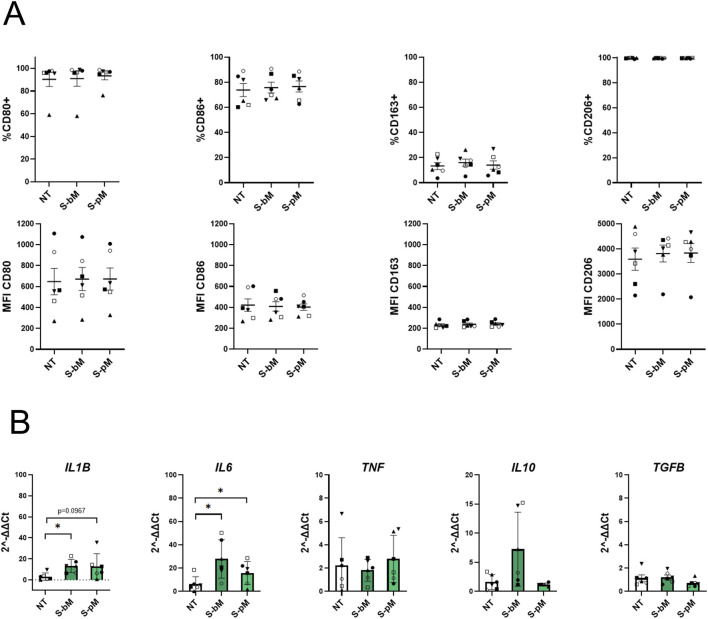
Effect of MenSC-derived secretomes on M1-like MDM polarization. Expression of M1-and M2-associated markers was analyzed in MDMs committed to an M1-like phenotype following treatment with S-MenSCs during the polarization phase. **(A)** Flow cytometry analysis of M1-associated markers (CD80 and CD86) and M2-associated markers (CD163 and CD206), represented as both the frequency of positive cells (% of cells) and the geometric mean fluorescence intensity (GeoMean). **(B)** RT-qPCR analysis of pro-inflammatory cytokines (*IL1B*, *IL6*, and *TNF*) and anti-inflammatory cytokines (*IL10, TGFB*). Data are presented as mean ± SEM. For n = 6 donors, normality was assessed by Shapiro–Wilk test; normally distributed data were analyzed by repeated-measures one-way ANOVA with Tukey’s *post hoc* test, and non-normal data by Friedman test with Dunn’s multiple comparisons. *, p < 0.05 and **, p < 0.005. NT, non-treated MDMs; S-bM, MDMs treated with secretome from basal MenSCs (S-bMenSC); S-pM, MDMs treated with secretome from IFNγ/TNFα-primed MenSCs (S-pMenSC).

**FIGURE 5:**
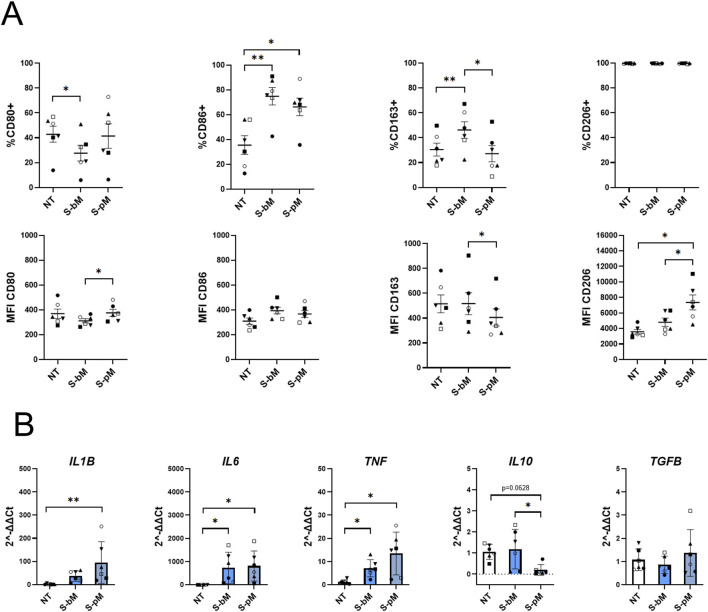
Effect of MenSC-derived secretomes on M2-like MDM polarization. Expression of M1-and M2-associated markers was analyzed in MDMs committed to an M2-like phenotype following treatment with S-MenSCs during the polarization phase. **(A)** Flow cytometry analysis of M1-associated markers (CD80 and CD86) and M2-associated markers (CD163 and CD206), represented as both the frequency of positive cells (% of cells) and the geometric mean fluorescence intensity (GeoMean). **(B)** RT-qPCR analysis of pro-inflammatory cytokines (*IL1B*, *IL6*, and *TNF*) and anti-inflammatory cytokines (*IL10, TGFB*). Data are presented as mean ± SEM. For n = 6 donors, normality was assessed by Shapiro–Wilk test; normally distributed data were analyzed by repeated-measures one-way ANOVA with Tukey’s *post hoc* test, and non-normal data by Friedman test with Dunn’s multiple comparisons. *, p < 0.05 and **, p < 0.005. NT, non-treated MDMs; S-bM, MDMs treated with secretome from basal MenSCs (S-bMenSC); S-pM, MDMs treated with secretome from IFNγ/TNFα-primed MenSCs (S-pMenSC).

After evaluating the secretome effects on monocytes, we further investigated how S-MenSCs may influence the phenotype MDMs during their polarization towards M1-and M2-like cells (Figures 4, 5, respectively). For MDMs already committed to an M1 phenotype, treatment with S-MenSCs did not alter the levels of CD80 and CD86 (Figure 4A). However, analysis of cytokine expression revealed a significant increase in *IL6* under both treatments, whereas *IL1B* was significantly elevated only following S-bMenSC treatment (Figure 4B). Under these circumstances, M2-associated markers exhibited minimal changes, with a tendency of *IL10* increase upon basal secretome treatment, and a certain reduction of *IL10* and *TGFB* levels with primed secretome. These findings might indicate that S-bMenSC treatment, by increasing *IL10*, may partially limit the induction of an inflammatory phenotype, whereas treatment with S-pMenSCs, in a context of pre-established M1 polarization, appears to keep polarization toward M1 phenotype (Figure 4). In contrast, MDMs committed to an M2-like phenotype showed a marked increase in M1-associated markers upon secretome stimulation. CD86 expression increased significantly under both S-bMenSC and S-pMenSC treatments (Figure 5A), while *IL6* and *TNF* were significantly elevated under both treatments, and *IL1B* increased significantly only with S-pMenSC (Figure 5B). M2-associated markers showed differential responses depending on the secretome type. The basal secretome (S-bMenSC) induced a significant increase in the proportion of CD163+ cells as compared to non-treated MDMs, whereas the primed secretome (S-pMenSC) significantly increased CD206 MFI and decreased *IL10* expression (Figure 5B). When comparing both secretomes, S-pMenSC significantly reduced CD163 and *IL10* levels relative to S-bMenSC, although both treatments increased CD206 expression. These findings suggest the basal secretome of MenSCs to induce a hybrid or prone profile of M2-committed MDMs, where pro-inflammatory markers are significantly upregulated (i.e., *IL6, TNF*), yet key M2 markers (*i.e.,* CD206, *IL10*) are retained, reflecting a balanced inflammatory response. Conversely, S-pMenSC treatment appears to partially shift M2-committed MDMs toward a more inflammatory state (*i.e.,* higher levels of *IL1B, IL6* or *TNF* expression, reduced levels of *IL10*), further supporting our observations on monocytes (Figure 3).

### 3.4 Effect of the MenSC-derived secretomes on macrophage phenotype modulation

**FIGURE 6:**
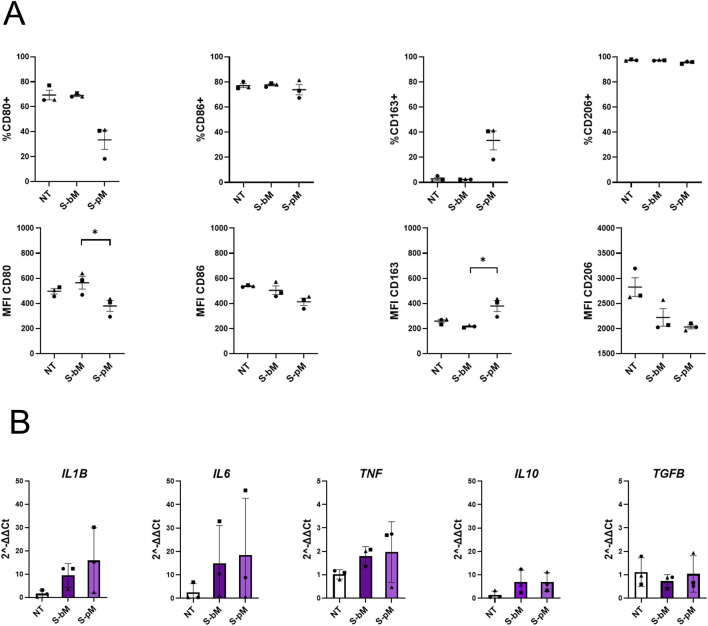
Effect of S-MenSCs in M1-like MDMs. *In vitro* differentiated and polarized M1-like MDMs were treated for 48 h with S-MenSC (100 μg/mL). **(A)** Flow cytometry analysis of M1-associated markers (CD80 and CD86) and M2-associated markers (CD163 and CD206), represented as both the frequency of positive cells (% of cells) and the geometric mean fluorescence intensity (GeoMean). **(B)** RT-qPCR analysis of pro-inflammatory cytokines (*IL1B*, *IL6*, and *TNF*) and anti-inflammatory cytokines (*IL10, TGFB*). Data represent mean ± SEM. Statistical significance was determined using the Friedman test with Dunn’s multiple comparisons (n = 3): *, p < 0.05; **, p < 0.005. (n = 3): *, p < 0.05 and **, p < 0.005. NT, non-treated MDMs; S-bM, MDMs treated with secretome released by menstrual blood-derived stromal cells in basal conditions (S-bMenSC); S-pM, MDMs treated with secretome released by menstrual blood-derived stromal cells pre-conditioned with IFNγ and TNFα (S-pMenSC).

**FIGURE 7:**
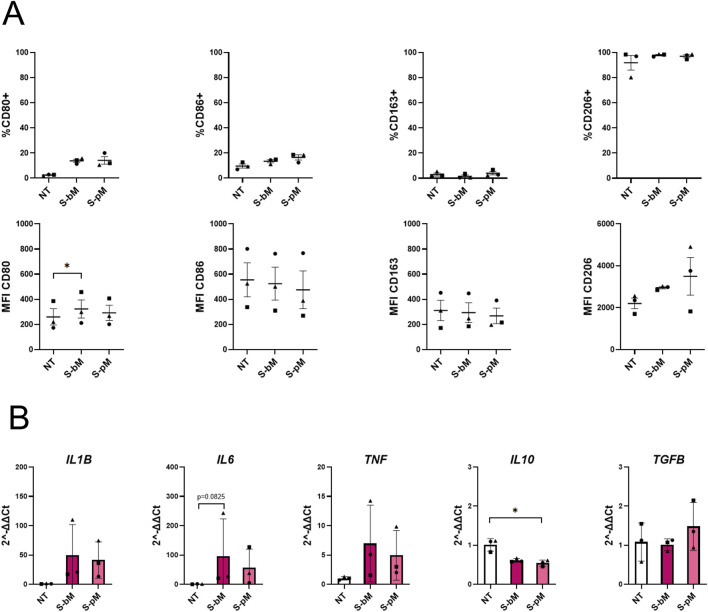
Effect of S-MenSCs in M2-like MDMs. *In vitro* differentiated and polarized M2-like MDMs were treated for 48 h with S-MenSC (100 μg/mL). **(A)** Flow cytometry analysis of M1-associated markers (CD80 and CD86) and M2-associated markers (CD163 and CD206), represented as both the frequency of positive cells (% of cells) and the geometric mean fluorescence intensity (GeoMean). **(B)** RT-qPCR analysis of pro-inflammatory cytokines (*IL1B*, *IL6*, and *TNF*) and anti-inflammatory cytokines (*IL10, TGFB*). Data represent mean ± SEM. Statistical significance was determined using the Friedman test with Dunn’s multiple comparisons (n = 3): *, p < 0.05; **, p < 0.005. (n = 3): *, p < 0.05 and **, p < 0.005. NT, non-treated MDMs; S-bM, MDMs treated with secretome released by menstrual blood-derived stromal cells in basal conditions (S-bMenSC); S-pM, MDMs treated with secretome released by menstrual blood-derived stromal cells pre-conditioned with IFNγ and TNFα (S-pMenSC).

Lastly, we investigated whether secretomes from S-MenSCs alter the phenotype of already polarized M1-and M2-like macrophages. To this end, M1-like as well as M2-like MDMs were polarized and, subsequently treated for 48 h with secretomes from basal or pro-inflammatory primed MenSCs in the absence of additional cytokines. Corresponding effects on the phenotype were assessed by FC and RT-qPCR on day 9. In M1-like MDMs (Figure 6), FC analyses showed that CD80 and CD86 were differentially modulated, with a significant reduction exclusively induced by S-pMenSCs (Figure 6A). For M2-associated markers, CD163 expression increased markedly with the primed secretome, whereas CD206 showed a non-significant trend for reduction. Regarding cytokine expression, a tendency for increased levels of the pro-inflammatory cytokines *IL1B*, *IL6*, and *TNF* was observed under both secretome treatments as compared to non-treated MDMs (Figure 6B). With respect to anti-inflammatory cytokines, we observed a general trend of increased *IL10* expression levels. Together, these findings suggest that treatment with S-bMenSCs maintains the existing pro-inflammatory M1 phenotype, while treatment with S-bMenSCs partially reduces M1-like subpopulations (*i.e.,* %CD80+) and increase M2-associated populations (*i.e.,* %CD163+), indicating a shift toward a more attenuated M1 phenotype. In M2-like MDMs (Figure 7), results indicated a slight increase in the M1-associated markers CD80 and CD86 upon secretome treatment, with CD80 showing a significant increase under S-bMenSCs (Figure 7A). For anti-inflammatory (M2) markers, CD206 exhibited non-significant upregulation trend under both treatments. Cytokine analyses showed a tendency toward increased pro-inflammatory cytokine expression (*IL1B*, *IL6*, and *TNF*) with both secretomes, whereas the anti-inflammatory cytokine *IL10* was significantly reduced under S-pMenSC treatment (Figure 7B). These findings suggest that treatment with both S-MenSCs may induce a shift in the phenotype of M2-like MDMs toward a more pro-inflammatory state. The strongest effect would be produced by the S-pMenSC, selectively downregulating M2-associated markers.

### 3.5 Protein profiling of S-MenSCs supports differential expression of macrophage polarization markers upon preconditioning

**FIGURE 8:**
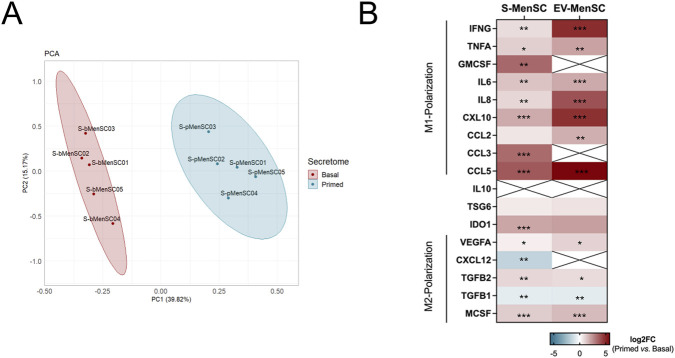
Comparative proteomic analysis of macrophage polarization markers in the secretome of MenSCs. **(A)** Principal Component Analysis (PCA) showing the clustering of S-MenSC samples based on their protein abundance profiles. **(B)** Heatmap displaying the relative abundance (log_2_ fold change) of macrophage polarization–associated proteins identified in S-pMenSC *vs*. S-bMenSC secretomes and their corresponding extracellular vesicle (EV) fractions. Red indicates increased abundance in primed conditions, blue indicates higher abundance in basal conditions, and crossed cells denote proteins not detected in the respective fraction. The dataset represents the intersection between the present proteomic analysis (total secretome) and the polarization markers described in our previous study on MenSC-EVs (de Pedro et al., 2023). Statistical significance was calculated by limma test: *, nominal *p value*<0.05; **, nominal p value<0.005; ***, nominal *p value*<0.0005. MenSC-EVs, extracellular vesicles derived from menstrual blood-derived stromal cells; S-bMenSCs, secretome released by non-treated, basal menstrual blood-derived stromal cells; S-pMenSCs, secretome released by menstrual blood-derived stromal cells preconditioned with IFNγ and TNF.

Taking into consideration the above findings from the *in vitro* analysis, our next aim was to explore proteinaceous differences between the two secretomes used for treatment in order to pinpoint molecules potentially contributing to the observed differences in monocyte and macrophage polarization. Firstly, we conducted a principal component analysis (PCA) on the proteomic data (Figure 8A) and found component 1 to explain approximately 40% of total variance, as well as clearly discriminating between sample types. Principal component 2 accounted for around 15% of the total variance, apparently representing inter-individual differences between MenSCs donors. Next, we identified differentially abundant proteins (DAPs) with a fold-change cutoff of |log_2_FC| ≥ 1 and a maximum adjusted p-value of 0.05, highlighting proteins with significant abundance differences between the primed and basal S-MenSC samples (Supplementary Figure S4A; Supplementary Table S5). This analysis identified 366 DAPs, of which 187 (51%) showed a significantly increased abundance in the primed condition, and 179 (49%) in the basal S-MenSC. Key abundant proteins in S-pMenSCs included CXCL9, MMP9, CCL5, CSF2, and ICAM1 (Supplementary Figure S4B), each known for their roles in immune response modulation and in polarizing macrophages towards an M1-like phenotype (Chen S. et al., 2023).

We subsequently selected 17 proteins, known to be directly associated with macrophage polarization (Chen S. et al., 2023) for further study (Figure 8B). Notably, all M1-polarization factors exhibited an increase in abundance in the secretome of MenSCs (S-MenSC) after preconditioning (primed) as compared to the basal condition. Among these markers, GMCSF, CCL3, and CCL5 showed the most statistically significant upregulation. When examining the corresponding EV fractions in our previous study (de Pedro et al., 2023), a similar trend was observed, except in the case of CCL3 and GM-CSF which were not detected (Figure 8B). These results indicate that these pro-inflammatory mediators are, at least partially, packaged into vesicles. For M2-polarization molecules, a differential pattern was observed when comparing basal and primed secretomes. In this case, MCSF, TGFB2, and VEGFA proteins showed increased abundance in primed conditions, while TGFB1 and CXCL12 showed reduced levels. As expected, IDO1 expression significantly increased in the secretome of MenSCs following pretreatment with IFNγ and TNFα (de Pedro et al., 2021); however, IL10 was unexpectedly undetectable, an observation similarly seen in EVs (Figure 8B).

## 4 Discussion

Macrophages are not only central to immune defense but also play a crucial role in the progression of numerous pathologies, including autoimmune disorders, inflammatory diseases like myocardial infarction and endometriosis, as well as cancer (Chen et al., 2024; Artemova et al., 2021). They are further essential for maintaining tissue homeostasis, promoting tissue repair, and resolving inflammation (Lu et al., 2021). Recent studies have shown that MSC-derived secretome promote the polarization of human macrophages toward an anti-inflammatory, reparative M2 phenotype, hinting at therapeutic potential (Peshkova et al., 2023; Holthaus et al., 2022; Chen Y. et al., 2023).

Among MSC sources, stromal cells derived from menstrual blood are particularly attractive due to both clinical and technical advantages. MenSCs are collected non-invasively and with little ethical concern, rendering them a comparatively viable alternative to other MSC sources. In addition, they exhibit a high proliferative capacity and extended *in vitro* lifespan, features that significantly enhance their handling and scalability for clinical and research applications (Lv et al., 2018; Bozorgmehr et al., 2020). Therapeutically, MenSCs have shown promising immunomodulatory and regenerative properties (Sanchez-Mata and Gonzalez-Munoz, 2021), mainly through their paracrine action. These attributes position MenSCs as strong candidates for cell-free therapies, particularly in the treatment of immune and inflammatory disease. However, the full extent of their modulatory effects on macrophage polarization remains underexplored. To address this, we investigated the *in vitro* effects of the secretome released by preconditioned or basal MenSCs on human monocytes and macrophages at different stages of polarization.

Firstly, we evaluated the therapeutic potential of basal and primed MenSCs via their secretome through a comprehensive bioinformatic analysis of the associated proteome. This approach revealed that the most abundant proteins in S-bMenSCs are involved in biological processes related to cellular communication, as well as key signaling pathways associated with the immune system, including both its innate and adaptive responses, and vesicular transport. Similarly, the most abundant proteins in S-pMenSCs were found to be involved in cellular communication and the regulation of angiogenesis, in addition to immune-related processes. These findings were consistent with our previous studies addressing the proteomic profile of EVs from MenSCs, exhibited distinct immunomodulatory and regenerative properties depending on the cell microenvironment (de Pedro et al., 2023).

After depicting the potential involvement of proteins of both secretomes in modulating the innate immune response, we further investigated their effects on macrophages. In our first experiment, we assessed the impact of the MenSC-derived secretome on monocyte differentiation. Our results demonstrated that both secretomes seem to promote macrophage phenotype characterized by preferential expression of pro-inflammatory markers, which strikingly diverges from most reports in the literature, namely the induction of a predominantly M2-like phenotype in naïve macrophages exposed to MSC-derived secretome (Deng et al., 2016; Vasandan et al., 2016; He et al., 2019). We hypothesize that this discrepancy may be related to unique characteristics of the MenSC-derived secretome. Notably, one possible explanation is the absence or undetectable levels of IL10, a key cytokine for promoting an anti-inflammatory macrophage phenotype (Holthaus et al., 2022).

We next examined the effect of S-MenSCs applied following polarization commitment by macrophages toward M1-or M2-like phenotypes by the surrounding environment. Importantly, the term “M2-like” used throughout the manuscript specifically refers to the M2c subtype, which is induced by IL10 and associated with immunoregulatory functions. Our results highlight that the both the basal and primed secretome seemed to promote polarization toward a pro-inflammatory M1 phenotype in a pro-inflammatory environment. While several studies have shown that MSC-derived secretomes typically promote anti-inflammatory polarization of macrophages (Peshkova et al., 2023; Dalirfardouei et al., 2019). These findings underscore once more the distinct biological activity of the MenSC-derived secretome in comparison to those reported for MSCs from other sources.

In contrast, in the context of anti-inflammatory conditions, the S-MenSC-derived secretome induced a partial shift toward an M1-like phenotype in MDMs, with distinct modulatory patterns depending on whether the secretome was basal or primed. The effect of the basal secretome was notably milder than that of the primed secretome. For instance, S-bMenSC modestly upregulated CD86 expression, whereas S-pMenSC prominently downregulated *IL10*.These observations underline that the modulatory effects of the S-MenSCs are due to different regulatory molecules, which in turn, are influenced by the macrophage polarization status. In support of these observations, our proteomic analysis revealed a differential enrichment of pro-inflammatory molecules in the MenSC-derived secretome and its EV fraction. Proteins such as IL8, TNF, IFNG, CCL3, CCL5, and GM-CSF (absent in EVs) were upregulated following priming, consistent with the induction of classical inflammatory pathways and an M1-like activation profile (Chen et al., 2018). Previous studies have shown that MSCs treated with TNF-α significantly increase the secretion of anti-inflammatory molecules such as TSG-6 (Torihashi et al., 2015), PGE2 (Ylostalo et al., 2012), and IL10 (Li et al., 2023), all of which are involved in promoting MSCs-mediated M2-type macrophage polarization. However, in our study, the MenSCs secretome induced a partial shift toward the M1 phenotype, rather than the expected shift to M2-like.

Finally, we explored the possible modulatory effect of MenSC-derived secretomes on fully polarized MDMs, both M1-and M2-like, which may have a further therapeutic application. In this context, treatment with S-bMenSCs maintained the M1-like phenotype in M1-MDMs and promoted a partial shift toward M1-like characteristics in M2-MDMs. These findings contradict previous reports showing that MSCs are likely to induce macrophages to transform into an anti-inflammatory phenotype (Lu et al., 2021). Interestingly, treatment with S-pMenSCs produced a partial phenotypic reversal: M1-like MDMs shifted toward an M2-like phenotype, while M2-like MDMs shifted toward an M1-like phenotype. This dual effect of S-pMenSCs may be attributed to increased abundance of immunomodulatory molecules such as IDO1 and TSG6, which are known to influence macrophage plasticity and promote a balance between pro- and anti-inflammatory responses (Peshkova et al., 2023). This enhanced capacity of S-pMenSCs to induce phenotypic changes suggests that preconditioning may prime the secretome to exert a more potent modulatory effect on macrophages than in the absence of preconditioning.

In summary, our findings reveal that both basal (S-bMenSCs) and preconditioned (S-pMenSCs) MenSC-derived secretomes have distinct and complex effects on macrophage polarization depending on the status of the target cells, highlighting in turn their potential use for immune modulation. In general, both secretomes seem to promote an M1 phenotype in pro-inflammatory environments and a hybrid M1/M2 or attenuated M1 phenotype in anti-inflammatory environments. Notably, S-pMenSCs play a key role in fully polarized MDMs, which may be capable of inducing a phenotype switch. These differences underscore the role of preconditioning in customizing MenSCs’ secretomes for targeted immunotherapeutic applications, enabling precise control of monocyte or macrophage responses. Future *in vivo* studies exploring these mechanisms may pave the way for new therapeutic strategies to manage inflammatory disorders and optimize immune modulation.

## 5 Limitations of the study

While this is a robust and well-controlled preliminary study, this work presents some limitations inherent to its design and exploratory nature that should be considered. First, all experiments were conducted exclusively under *in vitro* conditions; therefore, the findings need to be validated in animal models to confirm their pathophysiological relevance and translational potential. As an exploratory study focused on basic research, only a limited number of phenotypic and functional markers were assessed, without a comprehensive characterization of all potential activation states. Additionally, the secretome-treatment was tested at a single concentration based on our previous work (de Pedro et al., 2021; de Pedro et al., 2023), so potential dose-dependent effects cannot be excluded.

Regarding other limitations, although extensive washing steps were performed during both secretome collection and repolarization assays to remove priming (IFNγ/TNFα) and polarization (GM-CSF/M-CSF) cytokines, their complete elimination was not experimentally verified. Based on the included negative control, we do not expect residual cytokines to have exerted a substantial effect; however, this possibility cannot be entirely ruled out.

Moreover, a serum-free medium supplemented with ITS was used (Supplementary Figure S2). While this approach minimizes variability associated with serum components, ITS, even at residual levels remaining after cellular consumption, may exert biological effects on monocytes and macrophages, potentially confounding the interpretation of some results. Notably, the proposed treatment encompasses not only the secretome itself but also its full collection procedure, which will be replicated in future experiments.

Lastly, endotoxin levels were not measured, nor was batch-to-batch variability assessed, as all experiments were conducted using a single, fully characterized secretome batch. These factors may introduce experimental variability and should be addressed in future validation studies.

## 6 Conclusion

Our study demonstrates that the MenSC-derived secretome, collected under both basal conditions, as well as following priming of the cells, significantly influences macrophage polarization, with important potential implications for therapeutic application. S-bMenSC show strong potential to promote immune adaptability by inducing a hybrid M1-M2 phenotype in anti-inflammatory contexts, rendering their secretome particularly promising for conditions that require a balance between inflammation and tissue repair, such as chronic wounds, fibrotic diseases, and autoimmune disorders. Conversely, S-pMenSC, while tending toward a pro-inflammatory M1 profile, also enhance CD206 expression, a marker linked to M2 polarization. This duality suggests their potential utility in conditions requiring controlled inflammation, such as cancer immunotherapy or acute infection. This functional duality highlights the potential of MenSC preconditioning as a strategy to fine-tune macrophage responses according to therapeutic needs. Together, these data, as part of an exploratory framework, expand our understanding of MenSC secretome–macrophage interactions and provide a basis for addressing their therapeutic relevance *in vivo*. Continued investigation will clarify their mechanisms of action and optimize their use in future immunomodulatory strategies.

## Data Availability

Raw proteomic data, code for data processing and analysis are shared at the ProteomeXchange Consortium with dataset identifier: PXD060067, via the MassIVE partner repository (https://massive.ucsd.edu/, MassIVE-ID: MSV000096909; doi:10.25345/C5NZ81269). Raw qPCR and FC data are available in the Zenodo repository under the following DOIs: 10.5281/zenodo.17522320, 10.5281/zenodo.17540731, 10.5281/zenodo.17541002, 10.5281/zenodo.17541055, and 10.5281/zenodo.1754155.
