# Supplementary material for: Menstrual blood-derived mesenchymal stromal cell secretome modulates macrophage polarization in a preconditioning-dependent manner
Source: Front Cell Dev Biol. 2026 Jan 22;13:1691010. doi: 10.3389/fcell.2025.1691010 (PMC12872789; doi:10.3389/fcell.2025.1691010)
Supplement: Supplementary file 2 [file DataSheet1.docx]

Supplementary Material

# Supplementary Tables and Figures

## Supplementary Tables (Spreadsheets)

- **Supplementary Table 1.** Top 20% most abundant proteins in the secretome of basal MenSCs (S-bMenSCs).
- **Supplementary Table 2.** Gene Ontology (GO) and Reactome enrichment analyses for the top 20% most abundant protein in secretome of basal MenSCs (S-bMenSCs).
- **Supplementary Table 3.** Top 20% most abundant proteins in the secretome of basal MenSCs (S-pMenSCs).
- **Supplementary Table 4.** Gene Ontology (GO) and Reactome enrichment analyses for the top 20% most abundant protein in secretome of basal MenSCs (S-bMenSCs).
- **Supplementary Table 5.** Quantitative analyses of proteins detected by label-free mass spectrometry analyses in secretome samples. Abundance ratio is expressed as log2 fold-change of the normalized intensity values between primed (S-pMenSC) and basal (S-bMenSC) secretomes. Nominal and FDR-adjusted p values calculated by limma t-test are shown. The corresponding volcano plot is shown in Supplementary Figure 4A. TRUE/FALSE indicates imputed and non-imputed values for statistical evaluation. Note that this table also contains the number of precursors (Np) detected in a background sample consisting on DMEM medium containing ITS (green column). Individual values of Np for basal (blue) and primed (red) replicates are also provided.

## Supplementary Figures


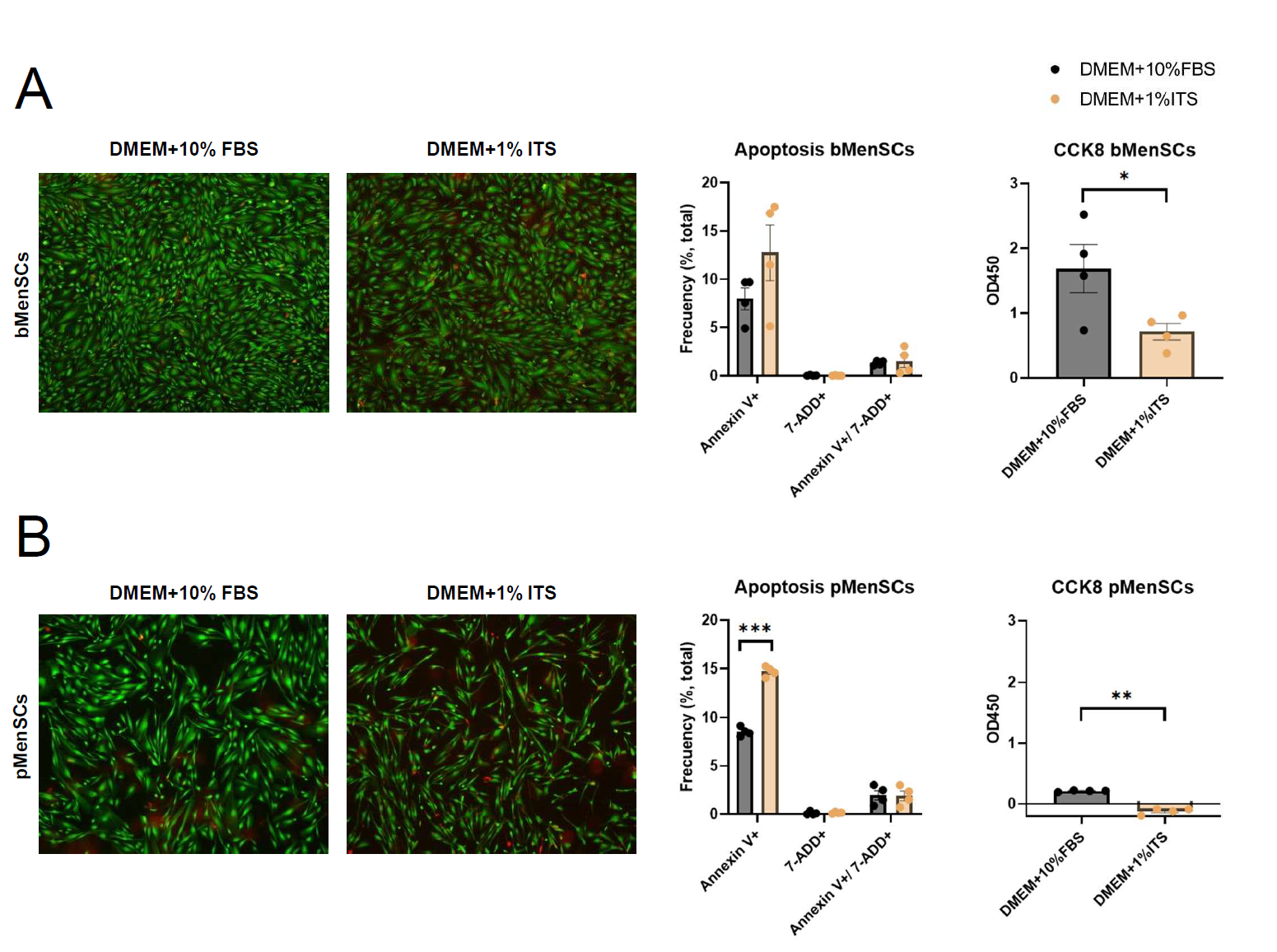


**Supplementary Fig.1** **Effect of ITS supplementation on basal and IFNγ/TNFα-primed MenSCs during secretome collection.** Basal and primed MenSCs were cultured in complete medium (DMEM + 10% FBS) or secretome-collecting medium (DMEM + 1% ITS). Representative Live/Dead images show live cells in green and dead cells in red. Bar graphs showed flow-cytometry quantification of apoptosis (left) and metabolic activity (CCK-8) (right) for both basal **(A)** and preconditioned **(B)** MenSCs. Grey bars: standard, FBS-containing medium; orange bars: serum-free, ITS-containing medium. Data represent mean ± SEM. Statistical significance was determined using Student’s t-test (n = 4). *, p < 0.05 and **, p < 0.005, ***, p< 0.0005.


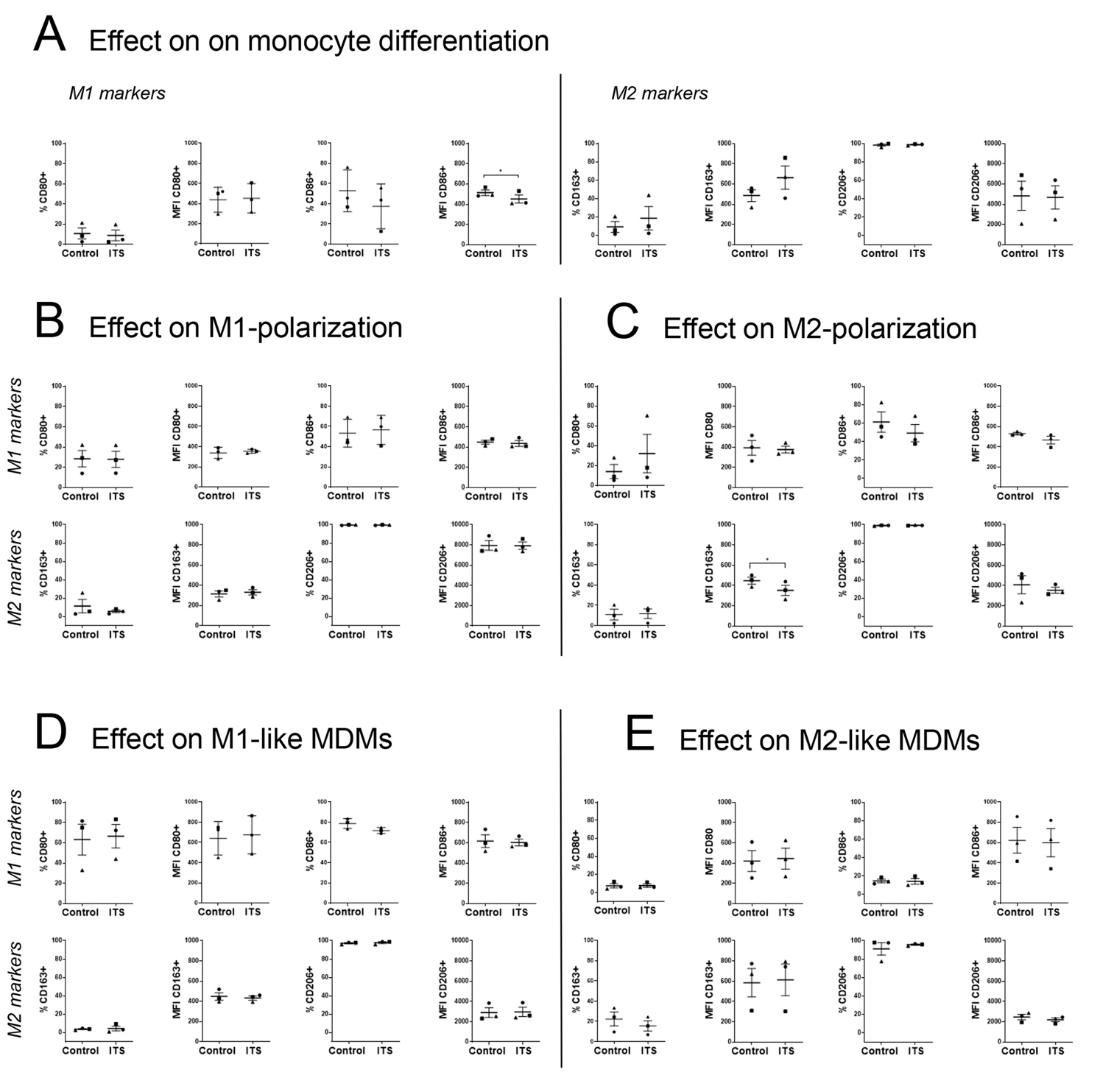


**Supplementary Fig.2 Evaluation of potential detrimental effects of secretome collection medium on monocytes and macrophages by flow cytometry.** To obtain the MenSC-derived secretome, the medium was replaced by serum-free medium supplemented with 1% ITS. The potential detrimental effect of ITS on the monocyte differentiation **(A)**, during M1- **(B)**, M2- **(C)** polarization, as well as on M1-like **(E)** and M2-like polarized MDMs **(F)** was evaluated by using a comparable volume of concentrated plain medium. Data are presented as mean ± SEM, complementing the results shown in Figures 3, 4, 5, 6 and 7. Statistical significance was tested by the Student’s t-test. (n = 3): *, p<0.05. NT, non-treated monocytes/MDMs; ∅ Med, monocytes/MDMs treated with a comparable volume of concentrated, plain secretome collection medium.


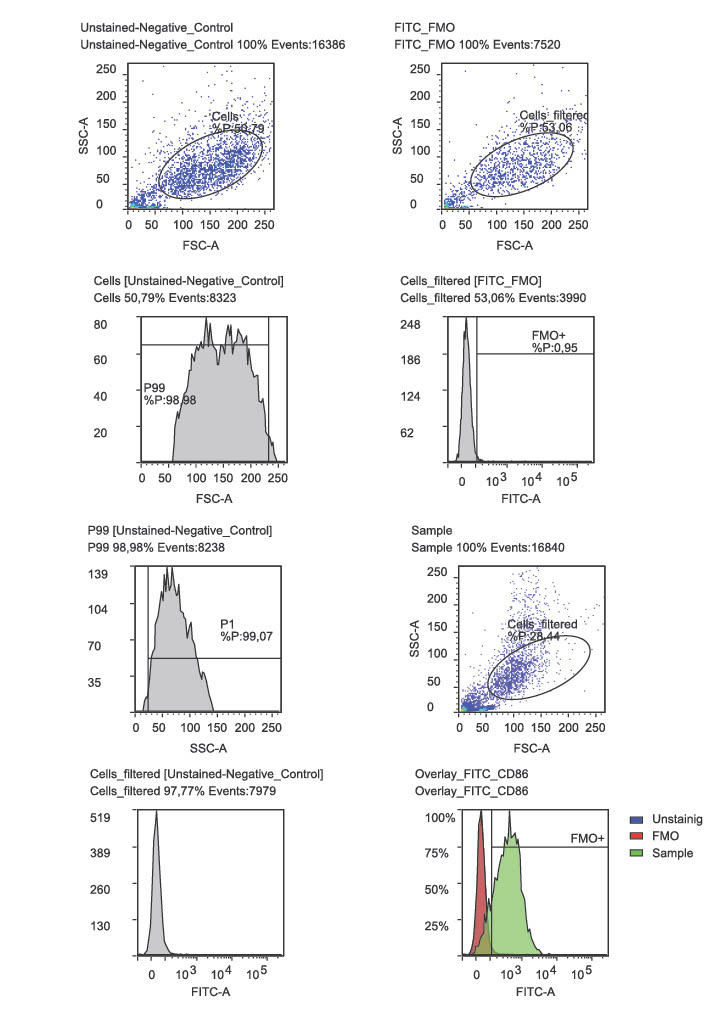


E

F

H

G

A

B

D

C

**Supplementary Fig.3 Gating strategy for flow cytometry analyses.** Panels show the sequential gating used to analyze macrophages. **(A)** Initial cell selection by FSC-A *vs.* SSC-A. **(B, C)** Filtering of extreme events (FSC-A > 99th percentile; SSC-A < 1st percentile). **(D)** Negative control (unstained) for FITC. **(E)** Selection of the same population in the FMO-FITC tube. **(F)** Determination of % positive cells using FMO threshold. **(G)** Filtered population in a fully stained sample. (H) Overlay of unstained, FMO, and fully stained FITC channel. FSA: Forward Scatter; SSC: Side Scatter; FMO: Fluorescence Minus One; FITC: Fluorescein Isothiocyanate.

**
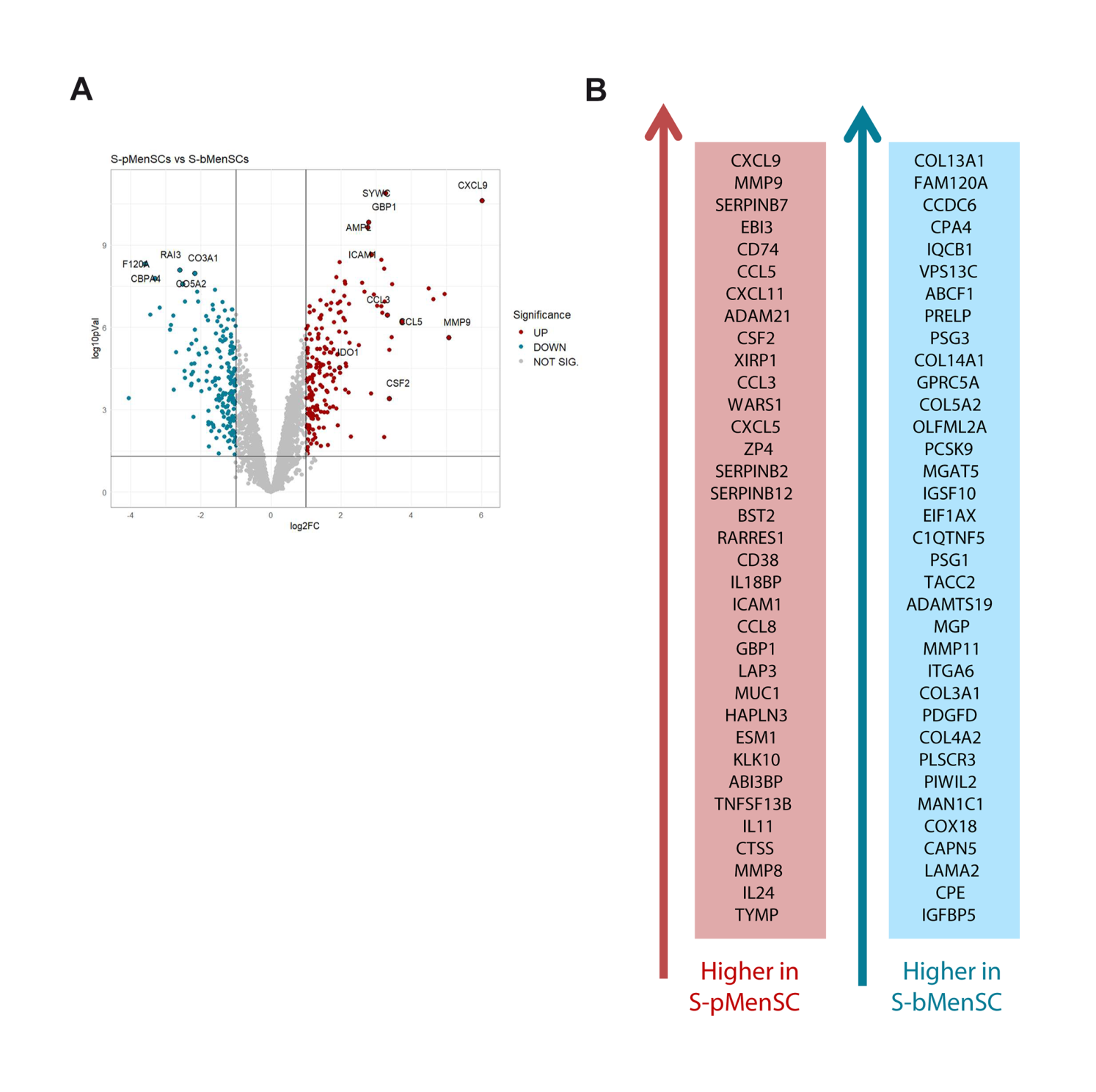
**

**Supplementary Fig.4 Differential proteome composition of the MenSC-derived secretome under basal and primed conditions.** **(A)** Volcano plot of differentially abundant proteins (DAPs) in the primed (S-pMenSC) *vs.* basal secretome (S-bMenSC) (n = 5 donors). Values indicate the log2fold-change (X-axis) and the -log10(nominal p value) (Y-axis). Significantly increased proteins in the primed (red dots, log2fold-change ≥ 1 and nominal *p* value < 0.05); or the basal secretome (blue dots, log2fold-change ≤ -1 and nominal p value < 0.05) are highlighted. Top 5 DAPs and other relevant proteins are individually labeled. Full list of proteins and corresponding statistics is depicted in Supplementary Table 5. **(B)** Top 35 DAPs with significant increase in primed (red) or basal (blue) samples. Statistical significance was calculated by limma test: *, p<0.05; **, p<0.005; ***, p<0.0005. S-bMenSCs, secretome released by non-treated, basal menstrual blood-derived stromal cells; S-pMenSCs, secretome released by menstrual blood-derived stromal cells preconditioned with IFNγ and TNFα.
